# Molecular Mechanism of STIL Coiled-Coil Domain Oligomerization

**DOI:** 10.3390/ijms241914616

**Published:** 2023-09-27

**Authors:** Mai Shamir, Freddie J. O. Martin, Derek N. Woolfson, Assaf Friedler

**Affiliations:** 1 Institute of Chemistry, The Hebrew University of Jerusalem, Safra Campus Givat Ram, Jerusalem 91904, Israel; mai.shamir@mail.huji.ac.il; 2 School of Chemistry, University of Bristol, Cantock’s Close, Bristol BS8 1TS, UK; f.martin@bristol.ac.uk; 3 School of Biochemistry, University of Bristol, Biomedical Sciences Building, University Walk, Bristol BS8 1TD, UK; 4 Bristol BioDesign Institute, University of Bristol, Cantock’s Close, Bristol BS8 1TS, UK

**Keywords:** STIL, coiled coil domains, oligomerization, peptides, protein interactions

## Abstract

Coiled-coil domains (CCDs) play key roles in regulating both healthy cellular processes and the pathogenesis of various diseases by controlling protein self-association and protein–protein interactions. Here, we probe the mechanism of oligomerization of a peptide representing the CCD of the STIL protein, a tetrameric multi-domain protein that is over-expressed in several cancers and associated with metastatic spread. STIL tetramerization is mediated both by an intrinsically disordered domain (STIL_400–700_) and a structured CCD (STIL CCD_718–749_). Disrupting STIL oligomerization via the CCD inhibits its activity *in vivo.* We describe a comprehensive biophysical and structural characterization of the concentration-dependent oligomerization of STIL CCD peptide. We combine analytical ultracentrifugation, fluorescence and circular dichroism spectroscopy to probe the STIL CCD peptide assembly in solution and determine dissociation constants of both the dimerization, (K_D_ = 8 ± 2 µM) and tetramerization (K_D_ = 68 ± 2 µM) of the WT STIL CCD peptide. The higher-order oligomers result in increased thermal stability and cooperativity of association. We suggest that this complex oligomerization mechanism regulates the activated levels of STIL in the cell and during centriole duplication. In addition, we present X-ray crystal structures for the CCD containing destabilising (L736E) and stabilising (Q729L) mutations, which reveal dimeric and tetrameric antiparallel coiled-coil structures, respectively. Overall, this study offers a basis for understanding the structural molecular biology of the STIL protein, and how it might be targeted to discover anti-cancer reagents.

## 1. Introduction

The SCL/TAL1 Interrupting Locus (*STIL*) gene is associated with the development of stem cell and T-cell leukemia [1]. The STIL protein plays a highly important role in mitosis by regulating centrosomal duplication. In the absence of STIL, tumor growth decreases [1,2]. STIL is over-expressed in multiple cancer types and is also associated with an increased metastatic potential in several cancer types [3,4,5]. On the other hand, it is not absolutely essential for the division of normal cells, making it an ideal anti-cancer drug target.

**Figure 1:**
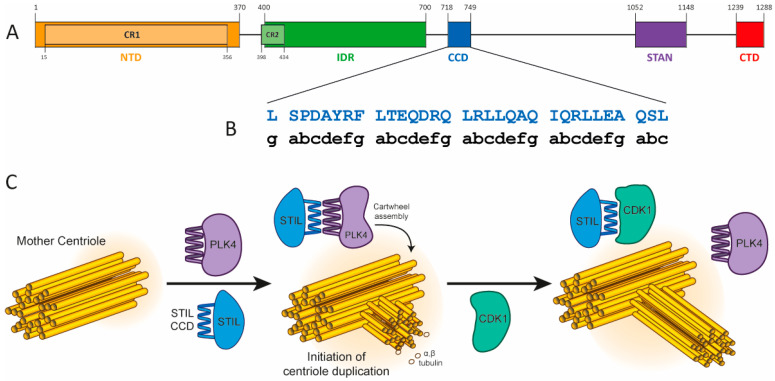
STIL domains and the CCD activity. (**A**) Domain structure of the STIL protein. See text for details. (**B**) STIL CCD amino-acid sequence with heptad repeats assigned from MARCOIL [30]. (**C**) STIL CCD interactions during centriole duplication. PLK4 (purple) recruits STIL to the mother centriole through its CCD (blue) and initializes the centriole duplication [9]. Then, at late G2 phase, CDK1 (green) competes with PLK4 on the binding to STIL CCD and by that prevents the STIL-PLK4 complex until the next cell cycle [10].

STIL is a multi-domain protein that contains both structured and disordered regions (Figure 1). The structured domains of STIL are the *N*-terminal domain (NTD, STIL_2–300_), and a coiled-coil domain (CCD, STIL_718–749_). The intervening region of STIL (STIL_400–700_) is intrinsically disordered (IDR, STIL_400–700_). (Figure 1A). At the late G1/G1-S transition, the serine\threonine kinase PLK4 recruits STIL through its CCD to the base of the mother centriole. This leads to a conformational change in PLK4, enabling its kinase activity and preventing its degradation [6,7,8,9]. Then, at late G2 phase and during the M phase, the CDK1 kinase is transiently expressed and competes with PLK4 for binding to the CCD of STIL, preventing PLK4 from recruiting STIL until mitosis is completed (Figure 1C) [10,11,12,13].

The CCD of STIL is necessary for its self-association [14,15]. Previously, we have shown that the oligomerization of STIL CCD peptide results in an increase in its thermal stability and is essential for STIL function *in vivo* [14]. STIL binds PLK4 as a monomer, forming a heterodimeric coiled coil with the PB3 domain of PLK4 [9]. On the other hand, STIL and its orthologous protein *Drosophila* Ana2 CCD (residues 193–229) form tetramers and this tetramerization is essential for centriole assembly [13,15]. However, the molecular mechanism by which the tetramerization regulates centriole assembly is unclear. Previously, we have shown that STIL also undergoes higher-order oligomerization through its IDR upon binding to Zn^2+^ ions together with a structural change of its NTD (residues 2–370, Figure 1A) [16]. Inhibition of STIL oligomerization through its CCD and/or IDR is a promising new potential target for cancer therapy. The physiological concentration of the STIL protein is constantly changing, depending on both expression levels and local concentration at different times and in different pathways during the cell cycle. Therefore, the precise cellular concentration of STIL is unknown.

CCDs are found in up to ≈10% of all eukaryotic proteins [17,18], where they are critical for regulating many processes in cells and are involved in the pathogenesis of various diseases [19]. The oligomerization of CCDs directs and defines the stoichiometry of both protein self-association and more-complex protein–protein interactions, as is the case with STIL (oligomerization and binding to PLK4) [20]. The sequences of CCDs usually comprise heptad repeats of hydrophobic amino acids (*h*), alternately spaced three and four residues apart with intervening polar residues (*p*), *hpphppp*. These are often denoted *a-b-c-d-e-f g* [21] with the hydrophobic side chains at positions *a* and *d* (For the heptad repeats of STIL CCD, see Figure 1B and Table 1. These helix–helix interactions are intimate being mediated by so-called “knob-into-holes” packing, which leads to both stability and specificity of CCDs defined by relatively short sequences, i.e., as few as ≈30 residues [22,23,24,25,26]. Specifically, the identity of the hydrophobic side chains at *a* and *d* largely determines the oligomeric state and topology of natural dimeric, trimeric, and tetrameric coiled coils, which can be exploited in CCD design and engineering [25,27,28,29].

Our previous studies of the isolated STIL CCD domain (STIL CCD peptide) have shown that a single Leu-to-Glu substitution at position 736, a d site of the predicted CC repeat in the STIL CCD peptide, leads to a decrease in its thermal stability [14]. Presumably, this is due to the disruption of the hydrophobic CC interactions upon mutation from the hydrophobic Leu to the charged Glu. Indeed, expression of the full-length L736E mutant in *Stil*−/− mouse embryonic fibroblasts completely disrupts the formation of centrosomes and cilia compared to WT STIL, which rescues this phenotype [14]. Other mutations in the CCD of STIL also lead to decreases in the thermal stability of the peptide, but the L736E mutation results in the most significant inhibitory effect on the activity of the full-length STIL protein *in vivo*. Therefore, this mutation was selected for further study reported here. L736 is also essential for the STIL CCD interaction with PLK4 [14], as shown in a structure of STIL CCD-PLK4 complex [9]. This competition between oligomerization and binding to PLK4 via the same CCD may indicate a regulatory function of the STIL CCD.

To better understand the molecular mechanism of STIL CCD oligomerization, the 32-residue CCD peptide was studied as an isolated domain [14]. Here, we report a detailed biophysical and structural study of this domain. The oligomerization of WT STIL CCD peptide is compared to the previously reported destabilizing L736E mutated peptide, which is monomeric in solution, and to three newly designed mutant CCD peptides: A722L, Q729L, and a double mutant of these two substitutions. Both substitutions stabilize the domain and all three mutants form tetramers. From solution–phase studies, we find that the dimerization constant of the WT STIL CCD peptide is ≈10 µM and the tetramerization constant is ≈70 µM. This oligomerization is cooperative and increases the thermal stability of the CCD peptide. In addition, we have used X-ray protein crystallography to determine structures of the dimeric mutant L736E and the newly designed stable tetrameric mutant Q729L.

## 2. Results

### 2.1. Quantifying the Oligomerization of WT STIL CCD Peptide

**Figure 2:**
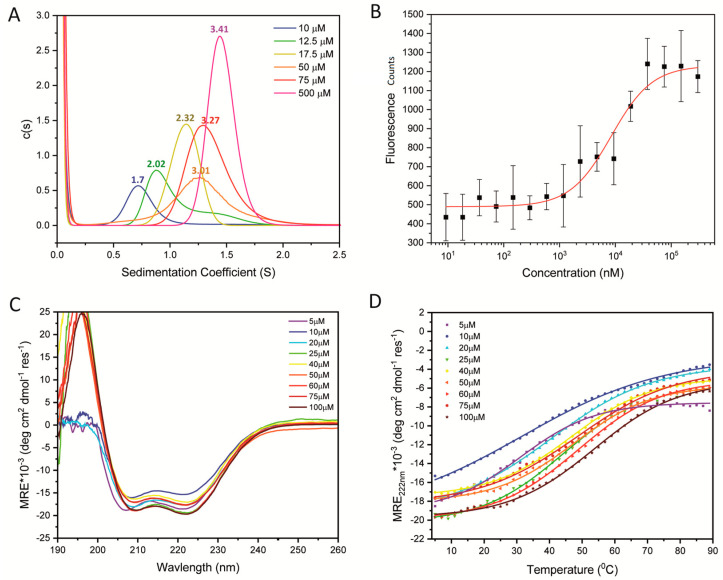
Characterization of WT STIL CCD peptide oligomerization. (**A**) AUC SV experiments fit to continuous c(s) distributions for 10, 12.5, 17.5, 50, 75 and 500 µM peptide concentrations in 25 mM sodium phosphate buffer, pH 6.8, ionic strength of 154 mM (NaCl). The resulting average number of monomers in the oligomer are given above each peak. (**B**) Fluorescence-based binding measurements of 5 µM labeled-WT CCD in the presence of serial dilutions of unlabeled CCD from 9 nM to 300 µM in 25 mM sodium phosphate buffer, pH 6.8, ionic strength of 154 mM (NaCl) with 5% DMSO. The measurement performed at room temperature. A K_D_ of 8 ± 2 µM was calculated from fitting to the Hill equation (red line). (**C**) CD spectra of WT STIL CCD at 5 °C and the different peptide concentrations given. (**D**) Thermal denaturation curves determined from CD signal at 222 nm for the same peptide concentrations in (panel **C**), and fit to the Boltzmann equation to determine T_m_ values. Conditions for all CD experiments: 25 mM sodium phosphate buffer, pH 6.8, 154 mM NaCl.

Previously, we and others [14,15] have shown by size-exclusion chromatography and multi-angle light scattering that the oligomerization of a STIL CCD peptide is concentration dependent. However, these studies were qualitative and the interaction was not quantified. To quantify the oligomerisation of the STIL CCD peptide, analytical ultracentrifugation (AUC) sedimentation velocity (SV) and sedimentation equilibrium (SE) experiments were performed (Figure 2A, Table 1 and Figures S4–S9, S18 and S19). AUC-SV experiments were conducted at several concentrations to explore how the oligomeric state changed with concentration in the low-to-high µM concentration range, and also to estimate the dissociation constants of the oligomerization of the STIL CCD peptide.

Consistent with previous studies [15], initial AUC-SV experiments indicated that the oligomeric state of the WT CCD changed with peptide concentration; for instance, an apparent dimeric species (2.0 × monomer mass) predominated at 12.5 μM, and higher-order species (3.4 × monomer mass) at 500 μM. To quantify the dissociation constants of the possible monomer-dimer and dimer-tetramer transitions, we used fluorescence-based binding measurements. Using micro-scale thermophoresis, fluorescence measurements were taken at increasing concentrations of WT CCD peptide in a fixed background of 5 µM fluorophore-labelled peptide. The addition of unlabeled WT CCD peptide, up to 300 µM, resulted in increased fluorescence indicative of a shift towards higher-order oligomers (Figure 2B). The fluorescence did not change above 75 µM unlabeled peptide, suggesting no change in the populations of different oligomers. Fitting the fluorescence data to the Hill equation provided a dissociation constant of 8 ± 2 µM and a cooperativity of 1.2 ± 0.3 (Figure 2B, Table S4). We interpret this as being for the monomer-dimer transition, and that the fluorescence measurements are insensitive to the higher-order oligomerization. Further examination of the AUC-SV measurements also showed that the threshold concentration for the monomer-dimer transition was ≈10 µM, and at 75 µM the apparent ratio of dimer and tetramer (3.27 × monomer mass) was similar to that at 500 µM peptide (3.4 × monomer mass) (Figure 2A). Additional fluorescence polarization-based measurements using the same STIL CCD peptide and different fluorophore resulted in a dissociation constant of 8.5 ± 0.4 μM and a Hill coefficient of 1.15 ± 0.07 (Figure S1B, Table S4). To probe the possible dimer-tetramer transition, we turned to AUC-SE measurements with starting concentrations of 5, 10, and 20 µM of STIL WT CCD peptide (Figures S18 and S19). The equilibrated data were fitted first to a model for a single ideal species, which provided a species 2.9 × monomer mass (Table 1). As this was between the molecular weights expected for dimer and tetramer, we refitted the data to a dimer-tetramer model, which provided K_D_ = 68 ± 2 µM. It should be noted that the dimerization constant of the WT peptide was measured in the presence of 5% DMSO and using a fluorophore-labeled peptide, while the tetramerization constant was measured without DMSO and unlabeled peptides. The addition of the label and the presence of the DMSO, which was unavoidable for making the labeled peptide soluble, may have affected the measured dimerization constant. Thus, we avoid directly comparing the values obtained by the two methods.

Next, we used circular dichroism (CD) spectroscopy to probe the secondary structures of these WT CCD peptide assemblies. Over the measured range of 5–100 µM and at 5 °C, the CD spectra changed little with peptide concentration (Figure 2C; HT traces and full CD spectra at different temperatures are shown in Figure S2). The spectra all had minima at 208 and 222 nm indicative of largely α-helical structures. From the high-concentration spectra we estimated ≈47% helix. To compare how the stabilities of these helical assemblies changed with concentration, we followed the changes in MRE_222_ with temperature over the same concentration range (Figure 2D, Table S1). Sigmoidal curves indicative of cooperative unfolding were apparent at all concentrations, indicating a cooperatively folded, oligomeric species. For of these transitions, the midpoints of the thermal transitions (T_M_) could be determined and increased from ≈31–53 °C (Table S1). The temperature-dependent CD measurements were performed to determine the oligomerization-dependent thermal stability of the CCD. The spectra were measured at 5 °C in order to serve as a starting point for these experiments. Combining the analysis of the peptide structure at different concentrations at a constant temperature (5 °C) and the denaturation measurement at the same concentrations, demonstrate that the change in T_m_ upon heating is mediated by oligomerization and not a change in structure.

Thus, we find that a peptide corresponding to the WT STIL CCD behaves as a complex mixture of monomeric, dimeric, and tetrameric species in the µM concentration range. Nonetheless, we have been able to disentangle this and measure apparent dissociation constants of ≈10 µM and ≈70 µM for the monomer-dimer and dimer-tetramer transitions, respectively. Moreover, the oligomeric species are cooperatively folded with mesophilic thermal stabilities.

### 2.2. Destabilising and Stabilising Mutations in the CCD Peptide of STIL

**Figure 3:**
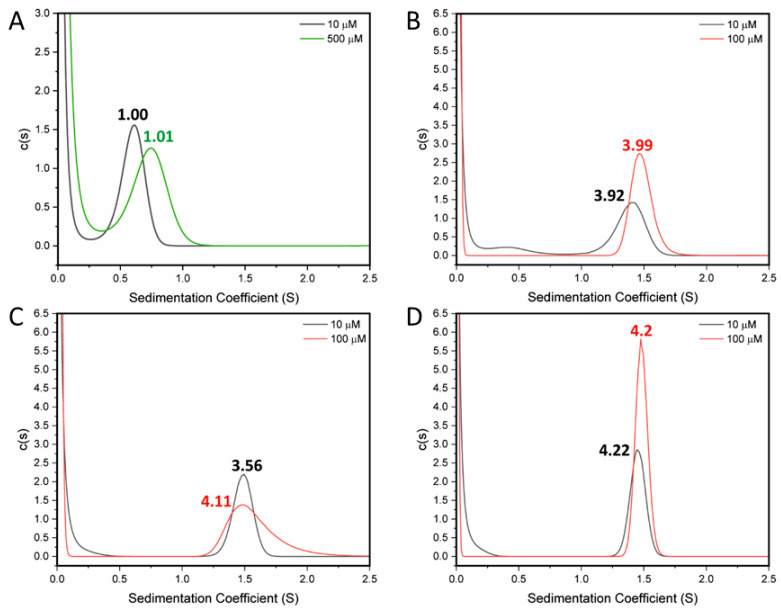
Characterization of the folding and oligomerization of mutant STIL CCD peptides–AUC analysis. AUC-SV data fit to continuous c(s) distributions for the (**A**) L736E, (**B**) A722L, (**C**) Q729L and (**D**) A722L + Q729L STIL CCD peptides. These were measured at 10 µM (black), 100 µM (red) and 500 µM (green) peptide concentrations. The L736E data returned a weight consistent with a monomer. The other mutants returned weights for tetramers. The calculated oligomer states are given above each peak.

**Figure 4:**
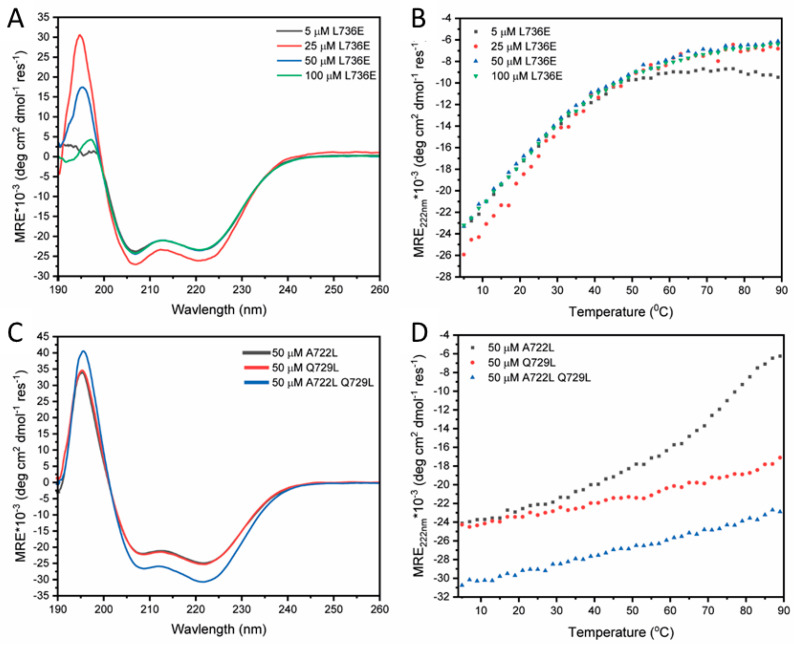
Characterization of the folding and oligomerization of mutant STIL CCD peptides–CD analysis. (**A**) CD spectra recorded at 5 °C, and (**B**) CD thermal denaturation curves fitted to the Boltzmann equation for T_m_ determination for L736E at different peptide concentrations. (**C**) CD spectra recorded at 5 °C and 50 µM peptide concentration for A722L (black), Q729L (red), and the double mutant, A722L + Q729L (blue). (**D**) CD thermal denaturation curves for the same samples in (panel **D**). Q729L and A722L + Q729L did not undergo denaturation, while A722L partially denatured with T_m_ of over 70 °C. Conditions: 25 mM sodium phosphate buffer pH 6.8, 154 mM NaCl.

As we have shown previously, the L736E mutant appears to disrupt hydrophobic interactions in STIL CCD peptide and tetramer formation, and it also decreases the melting temperature of the CCD peptide. This substitution also disabled the *in vivo* activity of the full-length STIL [14]. AUC-SV measurements at 10 µM and 500 µM of the CCD_718–749_-L736E peptide (Table 1) returned an oligomeric state of 1 × monomer mass (Figure 3A). Fluorescence binding measurements of this peptide under the same conditions as used for the WT peptide indicated no binding or oligomerization (Figure S1). CD spectroscopy showed that the peptide was helical, and this folding showed no concentration dependence (Figure 3B). The mutation did not disrupt the helical structure of the peptide. No detectable change in the Tm at different L736E concentrations was observed (Figure 4B, Table S2). These data are all consistent with the L736E mutation destabilizing the proposed oligomerization of the CCD peptide leading to a folded monomer in solution without the additional stability measured for the WT CCD.

To improve the stability of the CCD peptide, and so explore possible fully stabilized helical states, we sought to optimize the coiled-coil repeat in this sequence. Two-point mutations (A722L and Q729L) and a double mutant of these (A722L + Q729L) were designed (Table 1). Both of these sites are predicted as *d* positions in the coiled-coil heptad repeats, and as leucine (Leu, L) predominates at these positions in natural coiled coils we changed the Ala (A) and Gln (Q) to that residue [31,32]. AUC-SV experiments at 10 µM for these mutants all returned solution-phase molecular weights consistent with tetramers, and at 100 µM the data and fits were even more robust (Figure 3B–D and Table 1). CD spectra of the three mutants at 50 µM and 5 ℃ showed that the helical structure was preserved, and that the double mutant was 81% helical (Figure 3C). Moreover, thermal denaturation measurements revealed the mutants to be stabilized over the WT as intended. A722L had a cooperative unfolding transition with a T_m_ of over 70 °C, and Q729L and the A722L + Q729L double mutant did not undergo thermal denaturation (Figure 3D). The accessible thermal unfolding transition for A722L allowed its concentration dependence to be determined. Similar to the WT, the thermal stability of A722L increased with peptide concentration (Figure S3, Table S3).

### 2.3. X-ray Crystal Structures of the L736E and Q729L Variants

**Figure 5:**
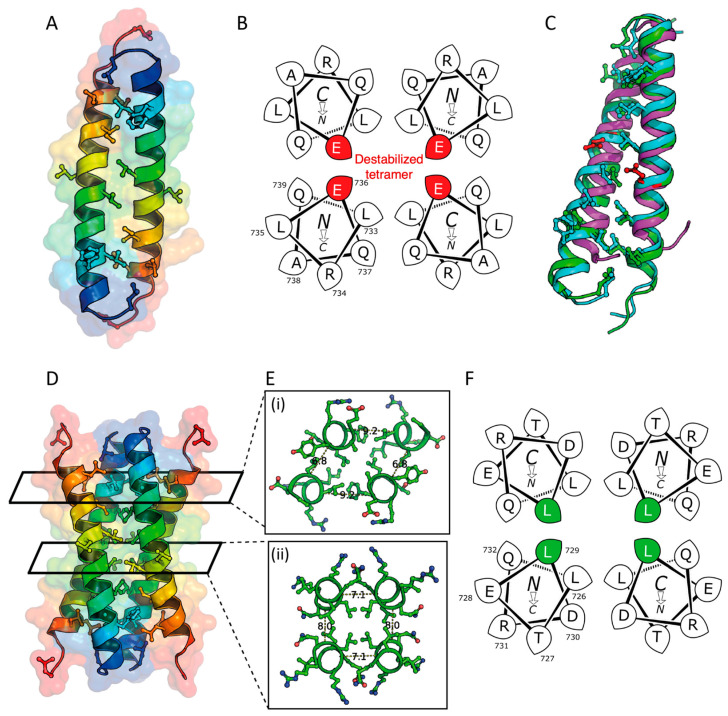
Structure characterization of STIL CCD L736E Dimer and Q729L Tetramer. (**A**) Structure of the antiparallel L736E dimer (PBD ID, 8oyk) with transparent surface colored from N → C termini as blue → red. (**B**) Helical-wheel projections for residues CCD_733–739_ from L736E with the glutamic acid of interest highlighted in red. (**C**) Alignment of STIL CCD L736E structure (green) to PDB entry 5lhw [15] (magenta, average RMSD = 6.1 ± 0.3 Å over an average of 47 Cα atoms), and to STIL CCD Q729L (cyan, average RMSD = 1.6 ± 0.5 Å over an average of 58 Cα atoms). Hydrophobic residues important for the dimerization and tetramerization are shown as balls and sticks. The point mutation is indicated in red. (**D**) Crystal structure of the antiparallel Q729L tetramer (PDB ID, 8oyl), shown as cartoon helices with transparent surface colored from N → C termini as blue → red, and with the hydrophobic residues as balls and sticks. (**E**) Slices of the Q729L structure at one end (**i**) and middle (**ii**), distances between the monomers shown as dashes, measured in Å. (**F**) Helical-wheel diagram of CCD726–732 Q729L, with the leucine of interest colored green. L736E was crystallized at 0.9 mM peptide, 50 mM Tris, pH 8.0, with 10% *v*/*v* MPD at 4 °C. Q729L was crystallized at 1.5 mM peptide, 50 mM cadmium chloride hemi(pentahydrate), 50 mM sodium acetate, and 15% *v*/*v* PEG 400, at pH 4.6 and 20 °C.

Despite the lack of assembly of the L736E variant, we were interested to explore its potential to assemble at very high peptide concentrations. Therefore, we attempted to crystallize the peptide. Crystals were obtained, diffraction data collected, and the structure was solved to a resolution of 1.67 Å using ARCIMBOLDO and α-helical search models [33]. The L736E peptide formed an antiparallel dimer in the crystal state. The structure has hydrophobic side chains in the tight dimer interface, which the program SOCKET2 [26] identifies as an antiparallel coiled coil with typical knobs-into-holes interactions (Figure 5A). However, there are fewer exposed hydrophobic residues that might be expected for the peptide to form a tetramer interface previously predicted by us and presented by others [15]. Helical-wheel projections for one heptad repeat of the L736E mutant indicate the potential disruption that would be caused by placing Glu-736 between the helices of the putative tetramer (Figure 5B). This may explain the monomeric state measured by the AUC-SV in solution. The remaining hydrophobic residues provide a small interface, disrupted by Glu-736, for crystal contacts in the unit cell and lattice (Figure S24). Alignment to of 5lhw, a previously determined structure of the STIL CCD, to the three biological assemblies of the STIL CCD L736E dimer show poor correlation between the hydrophobic residues important for the dimerization with an RMSD of 6.1 ± 0.3 Å (over an average of 47 Cαs) (Figure 5C, green), while the RMSD between the three biological assemblies is 1.5 ± 0.5 Å (over an average of 59 Cαs).

We also solved a structure of Q729L to 1.66 Å resolution. This revealed an anti-parallel tetramer, which we describe as a dimer of dimers (Figure 5D–F) [28]. The interaction between the monomers in the dimer is tighter than the interactions between the two dimers. Likewise, the packing in central portion of the tetrameric bundle is tighter than the ends (Figure 5E). Aligning the Q729L dimer to the L736E dimer resulted in mean RMSD of 1.6 ± 0.5 Å (over an average of 58 Cα atoms), indicating that the dimeric and tetrameric states are more structurally related than the biological assembly of the previously determined structure of the STIL CCD (Figure 5C, cyan).

## 3. Discussion

The results of the current study reveal a detailed molecular mechanism of the STIL CCD oligomerization. Specifically, for the first time, we measure the dissociation constants for both the dimerization and tetramerization of STIL CCD peptide. And we present X-ray crystal structures for the likely dimeric and tetrameric states.

### 3.1. The Role of STIL CCD Oligomerization during Centriole Duplication

STIL functions as a scaffold protein by interacting with multiple partners building the cartwheel complex during the formation of the new centriole [34]. This scaffolding requires binding flexibility as reported for other scaffold proteins [35,36]. The CCD of STIL binds both CDK1 and PLK4, at different times during the cell cycle [15]. The interaction of STIL CCD with the PLK4-PB3 domain (residues 884–970) is mediated by the same hydrophobic residues at the *d* positions, which are also responsible for STIL CCD oligomerization [9,14]. STIL CCD and PLK4-PB3 (residues 884–970) form a dimer, as shown in the solved structure of the complex, with K_D_ of 280 ± 60 nM as measured by isothermal titration calorimetry [9,15]. This value is an order of magnitude stronger than the 8 ± 2 μM K_D_ measured here for STIL CCD peptide dimerization. This suggests that the competition between oligomerization of STIL through its CCD and the CCD-mediated interactions of STIL with PLK4-PB3, which occur in the same biological process, is controlled by additional interactions in the context of the full-length STIL protein. Other STIL domains (such as the IDR) are indeed also involved in oligomerization and in interactions with other proteins. This cannot be observed in a study at the level of isolated domains, as performed here.

### 3.2. Destabilizing and Stabilizing Mutants of STIL CCD Peptide Leads to Monomeric and Tetrameric Oligomeric States, Respectively

The L736E mutation, found to be monomeric in the study presented here, is known to completely disrupt the activity of full length STIL *in vivo* [14]. The monomeric nature of the inactive L736E mutant indicates that changes in fluorescence observed in binding measurements of the WT CCD peptide are indeed oligomerization dependent. In addition, the observation that the T_m_ values change with the concentration in the WT but not in the monomeric STIL L736E indicates that the concentration-dependent stability of the WT peptide is attributed mainly to the formation of oligomers. Based on our understanding of coiled coils [31], two *d* positions in the CCD sequence–A722L and Q729L–were chosen for substitution to obtain a stabilizing effect. CCD mutants bearing these two substitutions as well as the double mutant A722L + Q729L formed very stable anti-parallel tetramers, which could not be dissociated under any experimental conditions. Thus, their K_D_s could not be measured.

Our structures of L736E and Q729L peptides reveal specific interactions within the CCD that mediate the oligomerization. The dimerization interface is tighter than the tetramerization interface with more hydrophobic residues mediating the interaction. This correlates with the 10 μM dimerization constant, indicating a stronger interaction compared to the dissociation measured for the tetramer (70 μM).

### 3.3. STIL CCD Activity during Centriole Duplication

**Figure 6:**
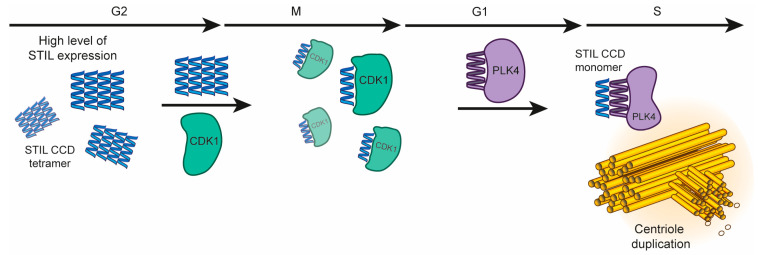
The role of STIL CCD. Schematic illustration of the activity of STIL CCD and its binding partner proteins during the cell cycle.

Here and previously [14], we have shown that the oligomerization of STIL CCD significantly stabilizes it and enables STIL activity in cells. This suggests that the oligomerization of STIL may have a regulatory role in keeping the expression levels of STIL at the correct concentration range. At high levels of expression this oligomerization can potentially keep STIL active and may prevent its degradation, while in further steps of the cell cycle, the lower oligomeric state STIL is more exposed to the APC/C destruction complex [3]. Accordingly, we suggest that the oligomerization of STIL and its interaction with other proteins are carried out in the following mechanism: (1) starting at the G2 stage, STIL level of expression is reaching a peak [37]. This results in the creation of tetramers that stabilize it. (2) Binding of STIL CCD to CDK1 during mitosis, which regulates a unique duplication of the centriole per one cell cycle. This interaction has not been fully characterized at the molecular level. (3) Then, recruitment of STIL monomer to the cartwheel complex by PLK4, at the late G1/G1-S, forming a hetero-dimeric coiled coil with PLK4-PB3 and initiation of the centriole duplication (Figure 6). The oligomerization of STIL may contribute to both the stability of the full-length protein and for ensuring its active subcellular concentration that is required for the centriole duplication process. This means that the dissociation of STIL tetramers into monomers near the mother centriole results in a higher subcellular concentration of active monomeric STIL, which can then bind to its other partners.

The fundamental study of the molecular mechanism of the oligomerization process of the CCD peptide of STIL, including the identification of the specific oligomerization constants and the structural analysis of both dimer and tetramer, is a prerequisite for designing STIL CCD inhibitors, which target both its oligomerization and its interactions with other proteins. Our new structures of STIL CCD peptide oligomers should serve as a basis for the rational design of inhibitors, and the dissociation constants that we have determined set the concentrations range at which such inhibitors must compete.

## 4. Materials and Methods

### 4.1. Peptide Synthesis, Labelling and Purification

All peptides were synthesized on a Liberty Blue microwave assisted peptide synthesizer (CEM) using standard Fmoc-SPPS chemistry on rink amide with DIC/Oxyma as coupling reagents in DMF and 20% piperidine in DMF as Fmoc deprotection reagent. Cleavage from the resin was performed with solution of 92% TFA, 5% water and 3% Tips. The cleaved peptides were purified on a reverse-phase high performance liquid chromatography (RP-HPLC) by preparative Waters 150Q LC HPLC system using a reverse-phase C18 preparative columns with a linear gradient range between 20–60% of ACN in TDW. LCQ Fleet ion Trap MS electrospray ionization mass spectrometer (ESI-MS) (Thermo scientific, Waltham, MA, USA) and analytical Merck–Hitachi HPLC were used to check the identity and verify the peptides purity. 7-(Diethylamino)coumarin-3-carboxylic acid (CAS-50995-74-9, Sigma Aldrich, St. Louis, MO, USA) fluorophore was coupled to the N terminus of the WT STIL CCD peptide before cleavage from the resin, using DIC/HOBt as activators. Concentration of all non-labelled CCD peptides were measured by the UV absorbance of Tyrosine that is part of the peptide sequence with extinction coefficient of 1490 M^−1^⋅cm^−1^ in aqueous solution. All peptides lyophilized and stored at −20 °C until use.

### 4.2. Crystallization

Lyophilized peptides were dissolved to a concentration between 5–10 mg/mL in deionized water (MilliQ) and vapor diffusion crystallization trials were performed using a Oryx 8 liquid handling robot (Douglas Instruments) into MRC-2drop 96 well plates. Droplets were prepared to a volume of 0.6 μL at a ratio of 1:1 and 1:2 of peptide stock to crystallization cocktail. Trials were setup using the Structure Screen 1 and 2 [38], JCSG+ [39], PACT Prem [40], Proplex [41,42], Morpheus [43] commercial screens (Calibre Scientific).

For conditions which produced crystals, the samples were cryoprotected in a prepared droplet of crystallization condition with 25% glycerol (*v*/*v*) and vitrified in liquid nitrogen.

X-ray diffraction data was collected at Diamond Light Source (BAGs: 17,212, 23,269, 26,335, at beamlines I03, I04, I04-1, and I24. Data was either processed through the xia2 dials [44] automated processing pipeline or reprocessed through the Dials User Interface [45] in the CCP4i2 package [46]. The datasets were phased using Arcimboldo [47] lite, ran in coiled-coil mode (this uses Phaser [48]). Initial models were then used in BUCCANEER [49] to add sidechains, and improve models and phases. Models were then improved using iterative cycles of COOT [50], Refmac [51], and PDB-REDO [52]. Images of the peptide structures were produced using PyMOL 2.0 (pymol.org/ (accessed on 12 May 2021).

### 4.3. AUC

Analytical ultracentrifugation (AUC) experiments were carried out using a Beckman Optima XL-A or XL-I analytical ultracentrifuge. Measurements were performed at 20 °C unless otherwise stated, using an An-50 or An-60 rotor, in Sodium Phosphate Buffer pH 6.8 (25 mM Na_2_PO_4_ with addition of 137 mM NaCl, pH measured using Jenway (TM) 3510 benchtop and adjusted using 1M HCl and 1M NaOH solutions). The partial specific volume ($\bar{\upsilon }$) for each peptide were calculated in Sedfit using values from Cohn and Edsall [53]. The buffer densities and viscosities were calculated using UltraScan III [54].

Sedimentation velocity (SV) experiments were performed in EPON 2-channel centerpieces with quartz windows or aluminium centrepieces with sapphire windows. The samples were centrifuged at 50 or 60 krpm where stated, with absorbance measurements taken from a radial range of 5.7–7.3 cm. 120 scans were taken at 5 min intervals, the data were fit in Sedfit to a continuous c(s) distribution model at a 95% confidence level. The frictional ration and baseline were floated, and Time-invariant and Radial-invariant noise were fit. Initially data were fit between a range of Svedberg ratio of 0–15, but this was reduced to 0–3 for all data as there were no larger species. The residuals for each individual fit are shown in Figures S14–S17 as a bitmap in which the color indicates the difference between the model fit and the raw data over the radial range to which the data were fitted. The scans are in vertical chronological order.

AUC sedimentation equilibrium (SE) experiments were run at multiple concentrations in a 6-Channel Epon charcoal-filled centerpiece. The samples were centrifuged at a range of speeds between 22–48 krpm. Absorbance scans across the radial range were recorded twice at each speed to ensure equilibration. Data were fitted to single, ideal species models or monomer-dimer equilibrium using Sedphat [55]. In total, 95% confidence limits were obtained via Monte-Carlo analysis of the obtained fits. The quality of the fit was evaluated using the *χ*^2^ parameter to observe which model best fit the data. Both fits of the data, where performed in Figures S18–S23 with their corresponding fit information.

### 4.4. CD Spectroscopy

CD spectra of the peptides were recorded using a J-810 spectropolarimeter (Jasco) in a 0.1 cm and 1 cm quartz cuvette for far-UV CD spectroscopy, in a spectral range of 190 nm to 260 nm. The buffer used in the CD measurements was 25 mM sodium phosphate buffer pH = 6.8 with ionic strength of 154 mM (adjected by NaCl). The thermal melting of all STIL CCD peptides were measured as described [14]. The graphs were fitted to Boltzmann equation in order to generate the T_m_. Normalization to mean residue ellipticity was performed. The 100% helicity was calculated (with normalization to the length of the peptide and the condition of the measurement) as MRE × 10^−3^ of −38 (Deg × cm^2^ × dmol^−1^ × res^−1^) [56]. According to this 100% parameter the % of helicity of each peptide was calculated (based on the MRE value that was measured in the same conditions, Table 1).

### 4.5. Fluorescence

In total, 5 μM of WT and L736E STIL CCD peptides labelled with 7-(Diethylamino)coumarin-3-carboxylic acid (CAS 50995-74-9, Sigma; *λ*_ex_ = 409 nm; *λ*_em_ = 473 nm) were incubated with increased concentrations of WT or L736E STIL CCD peptides in 25 mM Na_2_PO_4_ buffer with IS of 154 mM (NaCl), 5% DMSO. The samples were loaded on capillaries (Nanotemper). Fluorescence measurements were performed at room temperature (24 °C) using Monolith NT.115 system (Nanotemper) with 90% Blue LED power. Analysis and fitting performed using Origin software.

## Figures and Tables

**Table 1:** STIL CCD peptides sequence and oligomeric states.

Peptide Name	Amino Acid Sequence and CC Register	MRE_222 nm_ (kdeg cm^2^ dmol^−1^ res^−1^) (% Helicity) *	T_m_ (°C) at 50 µM	AUC Oligomeric State (Starting Peptide Concentration(s))		Oligomeric State (at 10 µM)	PDB ID
	*g abcdefg abcdefg abcdefg abcdefg abc*			SV (10 µM)	SE		
STIL CCD_718–749_ WT	L SPDAYRF LTEQDRQ LRLLQAQ IQRLLEA QSL-NH_2_	−17.6 (47%)	55.9 ± 0.5	1.7	2.9 ± 0.04 (5, 10, 20 µM)	Mixture of monomers and dimers	
STIL CCD_718–749_ L736E	L SPDAYRF LTEQDRQ LRL**E**QAQ IQRLLEA QSL-NH_2_	−23.3 (62%)	10 ± 2	1.00	0.79 ± 0.08 (500 µM)	Monomer	8oyk
STIL CCD_718–749_ A722L	L SPD**L**YRF LTEQDRQ LRLLQAQ IQRLLEA QSL-NH_2_	−24.9 (66%)	>70	3.92	3.51 ± 0.02 (5, 10, 20 µM)	Tetramer	
STIL CCD_718–749_ Q729L	L SPDAYRF LTE**L**DRQ LRLLQAQ IQRLLEA QSL-NH_2_	−25.3 (67%)	>95	3.56	3.47 ± 0.05 (5, 10, 20 µM)	Tetramer	8oyl
STIL CCD_718–749_ A722L Q729L	L SPD**L**YRF LTE**L**DRQ LRLLQAQ IQRLLEA QSL-NH_2_	−30.7 (81%)	>95	4.22	3.24 ± 0.03 (5, 10, 20 µM)	Tetramer	

* CD measured at 50 µM, 5 °C.

## Data Availability

Not applicable.

## Supplementary Materials

The following supporting information can be downloaded at: https://www.mdpi.com/article/10.3390/ijms241914616/s1.
