# Supplementary material for: Molecular Mechanism of STIL Coiled-Coil Domain Oligomerization
Source: Int J Mol Sci. 2023 Sep 27;24(19):14616. doi: 10.3390/ijms241914616 (PMC10572602; doi:10.3390/ijms241914616)
Supplement: Supplementary file 1 [file ijms-24-14616-s001.zip › ijms-2593990-supplementary.pdf]

Molecular Mechanism of STIL Coiled-Coil Domain Oligomerization

Mai Shamir [1,†], Freddie J. O. Martin [2,†], Derek N. Woolfson [2,3,4,*] and Assaf Friedler [1,*]

[1] Institute of Chemistry, The Hebrew University of Jerusalem, Safra Campus Givat Ram, Jerusalem 91904, Israel; mai.shamir@mail.huji.ac.il

[2] School of Chemistry, University of Bristol, Cantock's Close, Bristol BS8 1TS, UK; f.martin@bristol.ac.uk

[3] School of Biochemistry, University of Bristol, Biomedical Sciences Building, University Walk, Bristol BS8 1TD, UK

[4] Bristol BioDesign Institute, University of Bristol, Cantock's Close, Bristol BS8 1TS, UK

[*] Correspondence: d.n.woolfson@bristol.ac.uk (D.N.W.); assaf.friedler@mail.huji.ac.il (A.F.)

[†] These authors contributed equally to this work.

Table of Contents

Section 1. Fluorescence Assays **3**

Section 2. Circular Dichroism **3**

Section 3. Analytical Ultracentrifugation **7**

Sedimentation velocity bitmaps 7

Sedimentation equilibrium experiments 11

Section 4. Crystallography **14**

Section 1. Fluorescence Assays

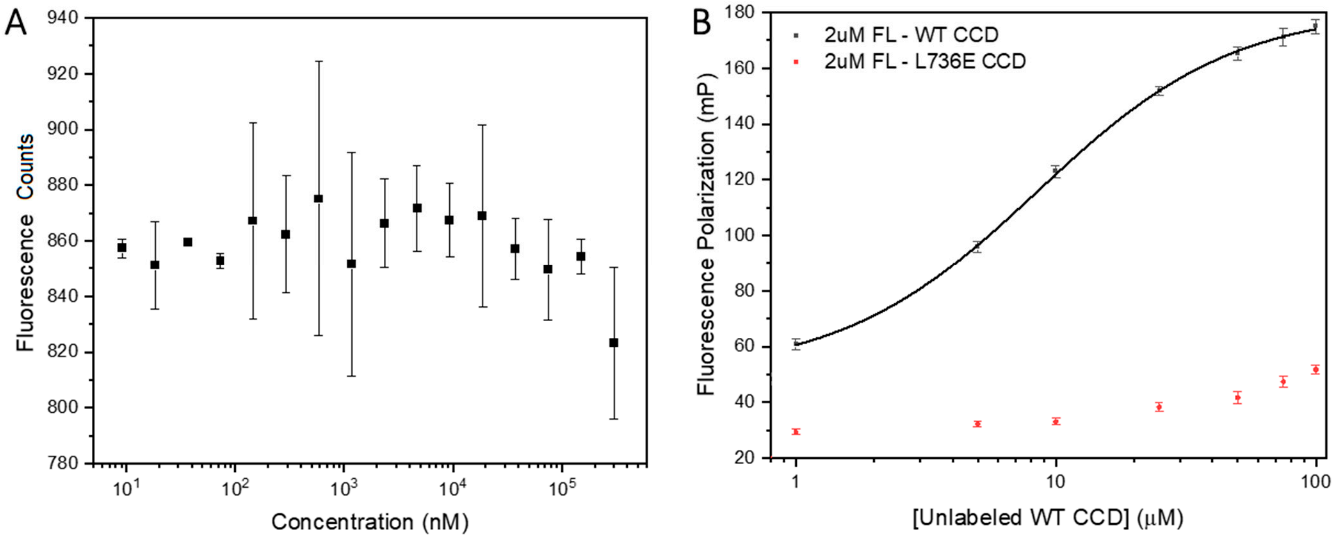

Figure S1: (A) Fluorescence-based binding measurement of 5 μM labeled-L736E CCD in the presence of serial dilutions of unlabelled L736E CCD from 9 nM to 300 μM in 25mM Na_2PO_4 pH 6.8, 5% DMSO. No binding was detected. (B) Florescence polarization measurements with different concentrations of WT STIL CCD (black) vs. L736E mutant (red). 2 μM fluorescein-labeled CCD and serial dilutions of unlabeled CCD in 25mM phosphate buffer with IS=100mM, pH=6.8 were measured. Kd of 8.5±0.4 μM was calculated from Hill equation.

Section 2. Circular Dichroism

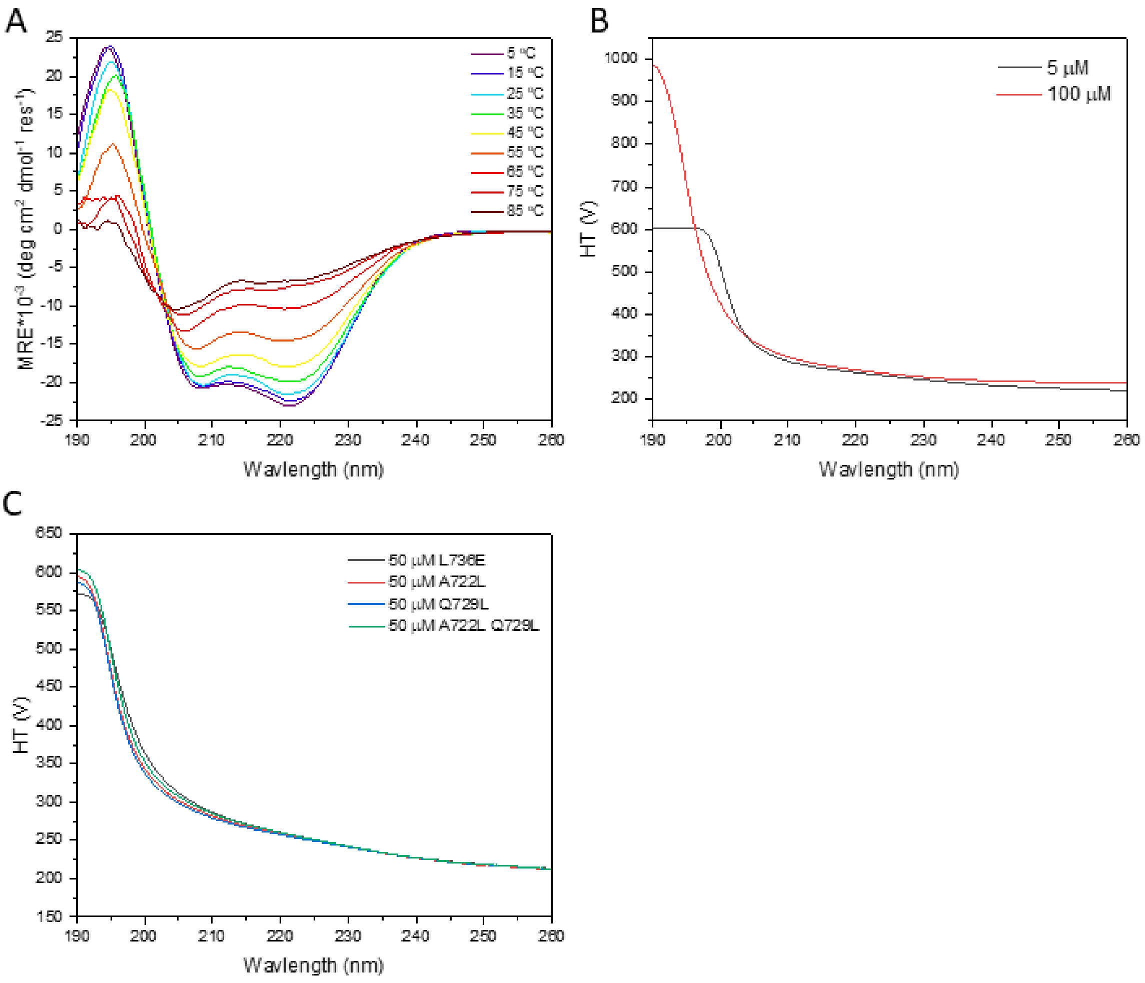

Figure S2: **Circular dichroism measurements of the CCD peptide.** (A) CD spectra of 50 µM WT STIL CCD at different temperatures. Iso-dichroic point is indicated at ~203nm; (B) CD-HT measurement of 5 µM and 100 µM WT STIL CCD measured using 1cm and 1mm cuvettes, respectively. (C) HT measurement of 50 µM STIL CCD mutants. All studies were performed in Sodium Phosphate Buffer pH 6.8.

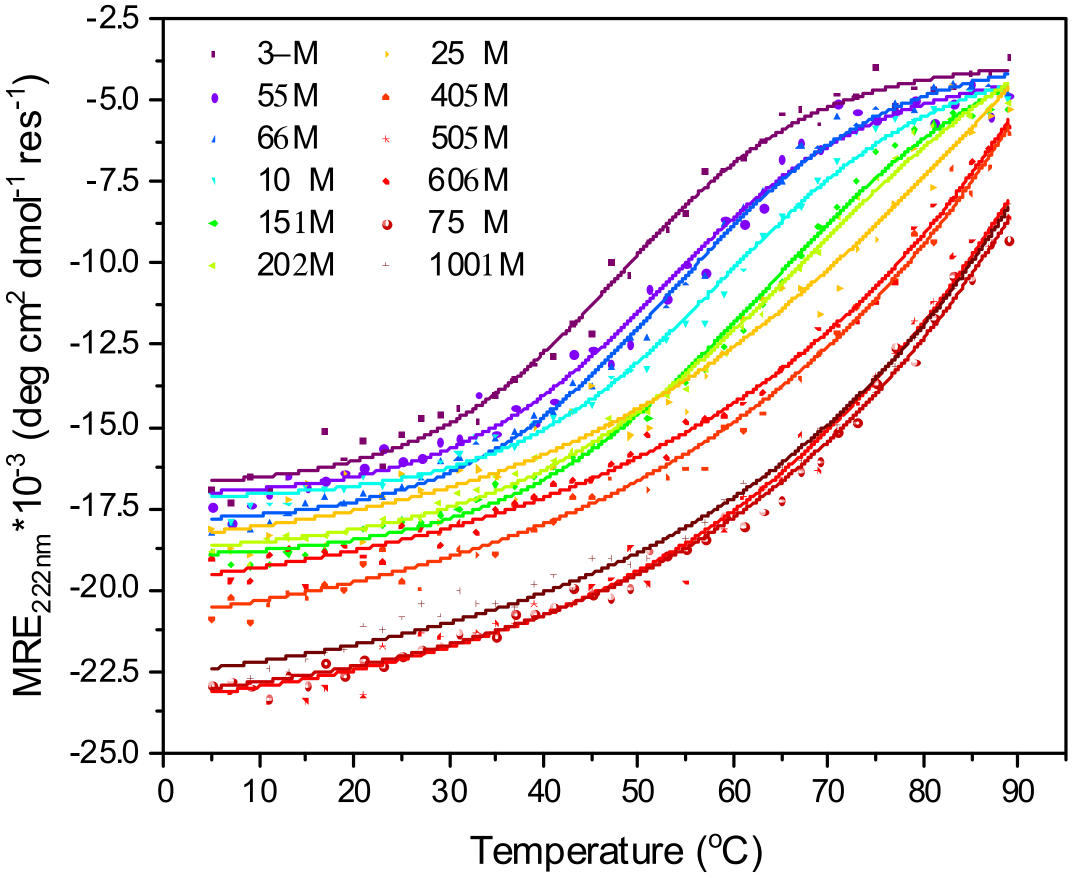

Figure S3:CD thermal denaturation of different concentrations of A722L in Sodium Phosphate Buffer pH 6.8. normalized to MRE, fitted to Boltzmann equation for the determination of T_m values.

Table S1: T_m values of the WT-CCD measured by CD:

Peptide concentration (μM)	Tm (°C)
5	31.1±0.8
10	31±1
20	40.6±0.3
25	44.2±0.3
40	46.6±0.2
50	47.4±0.3
60	48.8±0.3
75	48.6±0.5

100	53.6±0.4

Table S2: T_m values of the L736E CCD measured by CD:

Peptide concentration (μM)	Tm (°C)
5	18±1
25	17±2
50	10±2
100	12±1

Table S3: T_m values of the A722L CCD measured by CD:

Peptide concentration (μM)	Tm (°C)
3	48.0±0.7
5	52.9±0.9
6	53.6±0.6
10	59.1±0.8
15	63±1
20	68±2

Section 3. Analytical Ultracentrifugation

Sedimentation velocity bitmaps

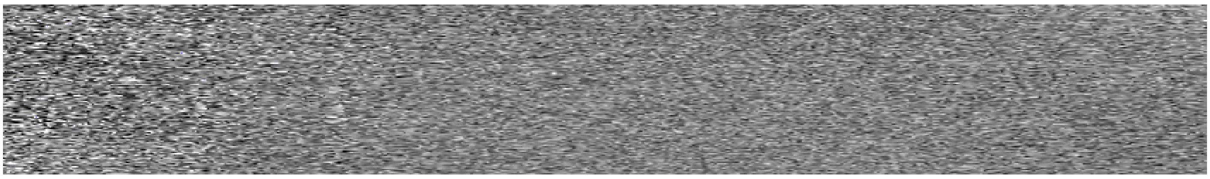

Figure S4: Residuals bitmap for the c(s) distribution fit to the AUC-SV data for the STIL-WT peptide ($\bar{v}$ = 0.737 cm^3 g^{-1}) at 10 µM shown in the Figure 2A. Data were recorded at 60 krpm returning fit parameter values of: sw = 0.757 S; sw(20,w) = 0.780 S; f/f0 =1.560; and mw = 6470 Da (1.7 × monomer mass) at 95% confidence level. Residuals are shown as a bitmap in which the greyscale shade indicates the difference between the fit and raw data, where black and white colors represent data that have extreme deviations from the fit.

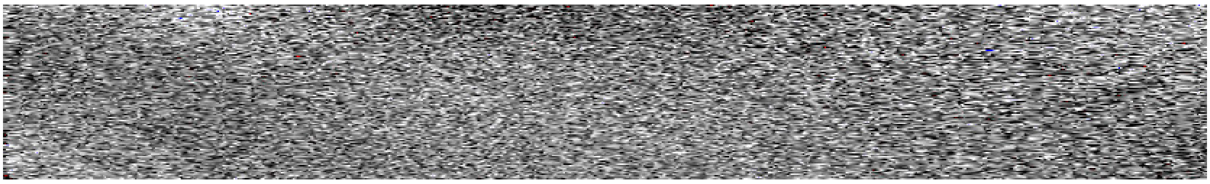

Figure S5: Residuals bitmap for the c(s) distribution fit to the AUC-SV data for the STIL-WT peptide ($\bar{v}$ = 0.737 cm^3 g^{-1}) at 12.5 µM shown in the Figure 2A. Data were recorded at 60 krpm returning fit parameter values of: sw = 1.054 S; sw(20,w) = 1.087 S; f/f0 = 1.259; and mw = 7717 Da (2.02 × monomer mass) at 95% confidence level. Residuals are shown as a bitmap in which the greyscale shade indicates the difference between the fit and raw data, where black and white colors represent data that have extreme deviations from the fit.

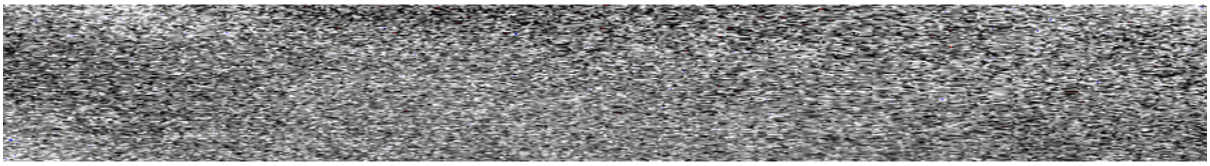

Figure S6: Residuals bitmap for the c(s) distribution fit to the AUC-SV data for the STIL-WT peptide ($\bar{v}$ = 0.737 cm^3 g^{-1}) at 17.5 µM shown in the Figure 2A. Data were recorded at 60 krpm returning fit parameter values of: sw = 1.130 S; sw(20,w) = 1.165 S; f/f0 =1.287; and mw = 8849 Da (2.32 × monomer mass) at 95% confidence level. Residuals are shown as a bitmap in which the greyscale shade indicates the difference between the fit and raw data, where black and white colors represent data that have extreme deviations from the fit.

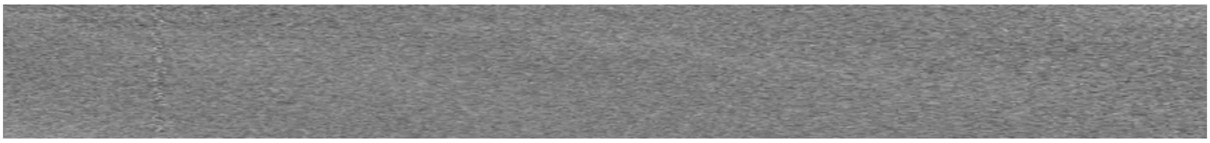

Figure S7: Residuals bitmap for the c(s) distribution fit to the AUC-SV data for the STIL-WT peptide ($\bar{v}$ = 0.737 cm^3 g^{-1}) at 50 µM shown in the Figure 2A. Data were recorded at 60 krpm returning fit parameter values of: sw = 1.268 S; sw(20,w) = 1.306 S; f/f0 =1.365; and mw = 11487 Da (3.01 × monomer mass) at 95% confidence level. Residuals are shown as a bitmap in which the greyscale shade indicates the difference between the fit and raw data, where black and white colors represent data that have extreme deviations from the fit.

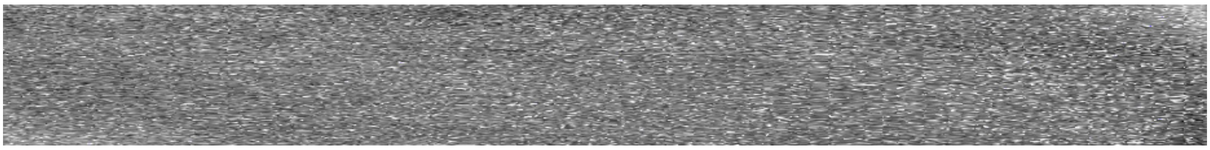

Figure S8: Residuals bitmap for the c(s) distribution fit to the AUC-SV data for the STIL-WT peptide ($\bar{v}$ = 0.737 cm^3 g^{-1}) at 75 µM shown in the Figure 2A. Data were recorded at 60 krpm returning fit parameter values of: sw = 1.321 S; sw(20,w) = 1.361 S; f/f0 =1.384; and mw = 12462 Da (3.27 × monomer mass) at 95% confidence level. Residuals are shown as a bitmap in which the greyscale shade indicates the difference between the fit and raw data, where black and white colors represent data that have extreme deviations from the fit.

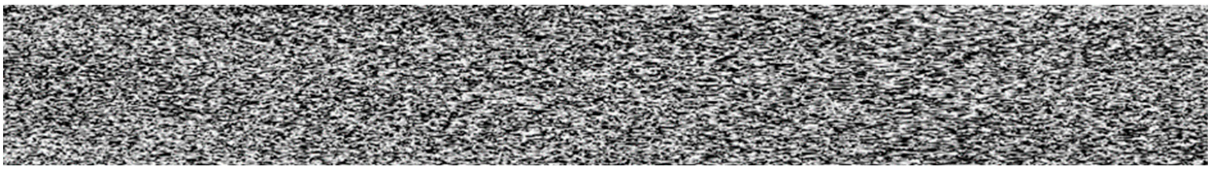

Figure S9: Residuals bitmap for the c(s) distribution fit to the AUC-SV data for the STIL-WT peptide ($\bar{v}$ = 0.737 cm^3 g^{-1}) at 500 µM shown in the Figure 2A. Data were recorded at 60 krpm returning fit parameter values of: sw = 1.459 S; sw(20,w) = 1.504 S; f/f0 =1.288; and mw = 12990 Da (3.41 × monomer mass) at 95% confidence level. Residuals are shown as a bitmap in which the greyscale shade indicates the difference between the fit and raw data, where black and white colors represent data that have extreme deviations from the fit.

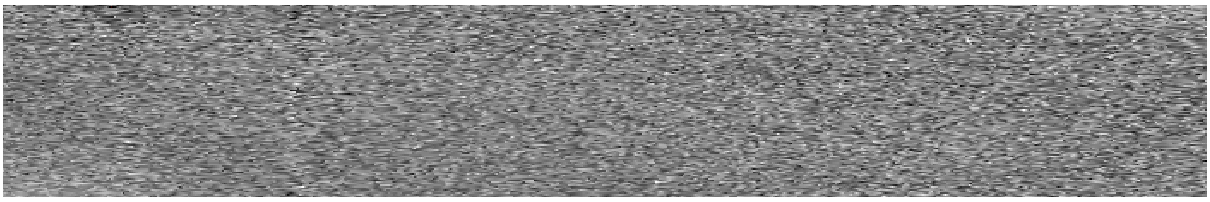

Figure S10: Residuals bitmap for the c(s) distribution fit to the AUC-SV data for the STIL-L736E peptide ($\bar{v}$ = 0.729 cm^3 g^{-1}) at 10 µM shown in the Figure 3A. Data were recorded at 60 krpm returning fit parameter values of: sw = 0.590 S; sw(20,w) = 0.608 S; f/f0 =1.460; and mw = 3849 Da (1.0 × monomer mass) at 95% confidence level. Residuals are shown as a bitmap in which the greyscale shade indicates the difference between the fit and raw data, where black and white colors represent data that have extreme deviations from the fit.

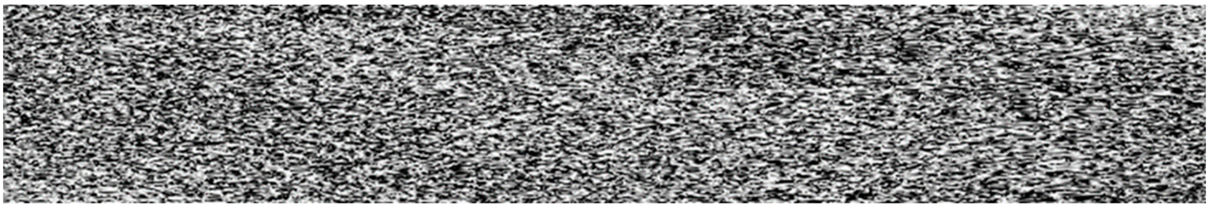

Figure S11: Residuals bitmap for the c(s) distribution fit to the AUC-SV data for the STIL-L736E peptide ($\bar{v}$ = 0.729 cm^3 g^{-1}) at 500 µM shown in the Figure 3A. Data were recorded at 60 krpm returning fit parameter values of: sw = 0.721 S; sw(20,w) = 0.742 S; f/f0 =1.201; and mw = 3871 Da (1.0 × monomer mass) at 95% confidence level. Residuals are shown as a bitmap in which the greyscale shade indicates the difference between the fit and raw data, where black and white colors represent data that have extreme deviations from the fit.

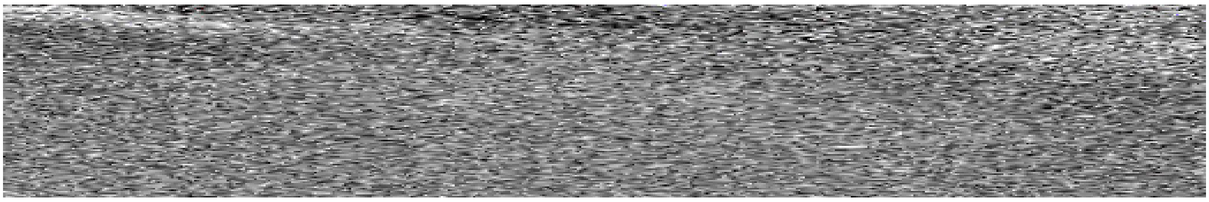

Figure S12: Residuals bitmap for the c(s) distribution fit to the AUC-SV data for the STIL-A722L peptide ($\bar{v}$ = 0.742 cm^3 g^{-1}) at 10 μM shown in the Figure 3A. Data were recorded at 60 krpm returning fit parameter values of: sw =1.384 S; sw(20,w) =1.428 S; f/f0 =1.458; and mw =15133 Da (3.9 × monomer mass) at 95% confidence level. Residuals are shown as a bitmap in which the greyscale shade indicates the difference between the fit and raw data, where black and white colors represent data that have extreme deviations from the fit.

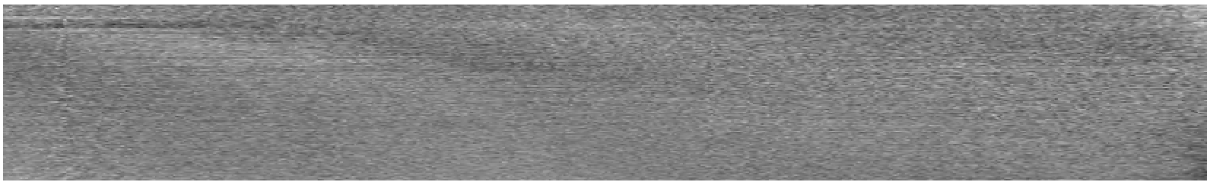

Figure S13: Residuals bitmap for the c(s) distribution fit to the AUC-SV data for the STIL-A722L peptide ($\bar{v}$ = 0.742 cm^3 g^{-1}) at 100 μM shown in the Figure 3A. Data were recorded at 60 krpm returning fit parameter values of: sw = 1.483 S; sw(20,w) = 1.514 S; f/f0 = 1.403; and mw 15385 = Da (4.0 × monomer mass) at 95% confidence level. Residuals are shown as a bitmap in which the greyscale shade indicates the difference between the fit and raw data, where black and white colors represent data that have extreme deviations from the fit.

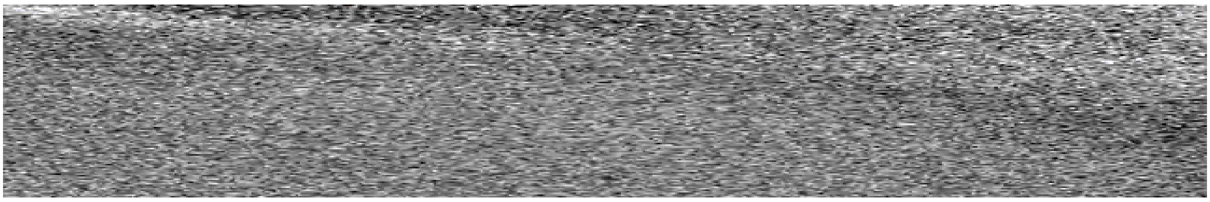

Figure S14: Residuals bitmap for the c(s) distribution fit to the AUC-SV data for the STIL-Q729L peptide ($\bar{v}$ = 0.746 cm^3 g^{-1}) at 10 μM shown in the Figure 3A. Data were recorded at 60 krpm returning fit parameter values of: sw = 1.484 S; sw(20,w) = 1.531 S; f/f0 = 1.250; and mw 13538 = Da (3.6 × monomer mass) at 95% confidence level. Residuals are shown as a bitmap in which the greyscale shade indicates the difference between the fit and raw data, where black and white colors represent data that have extreme deviations from the fit.

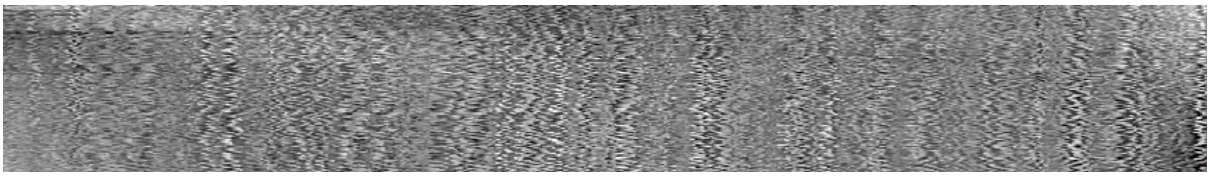

Figure S15: Residuals bitmap for the c(s) distribution fit to the AUC-SV data for the STIL-Q729L peptide ($\bar{v}$ = 0.746 cm^3 g^{-1}) at 100 μM shown in the Figure 3A. Data were recorded at 60 krpm returning fit parameter values of: sw = 1.573 S; sw(20,w) = 1.622 S; f/f0 = 1.323; and mw 15867 = Da (4.1 × monomer mass) at 95% confidence level. Residuals are shown as a bitmap in which the greyscale shade indicates the difference between the fit and raw data, where black and white colors represent data that have extreme deviations from the fit.

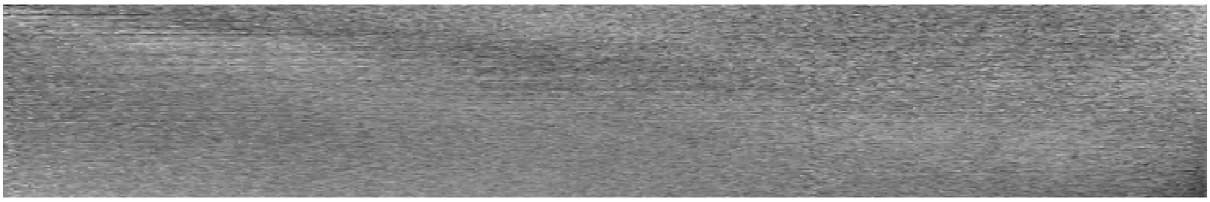

Figure S16: Residuals bitmap for the c(s) distribution fit to the AUC-SV data for the STIL-A722L-Q729L peptide ($\bar{v}$ = 0.751 cm^3 g^{-1}) at 10 µM shown in the Figure 3A. Data were recorded at 60 krpm returning fit parameter values of: sw = 1.452 S; sw(20,w) = 1.499 S; f/f0 = 1.411; and mw 16229 = Da (4.2 × monomer mass) at 95% confidence level. Residuals are shown as a bitmap in which the greyscale shade indicates the difference between the fit and raw data, where black and white colors represent data that have extreme deviations from the fit.

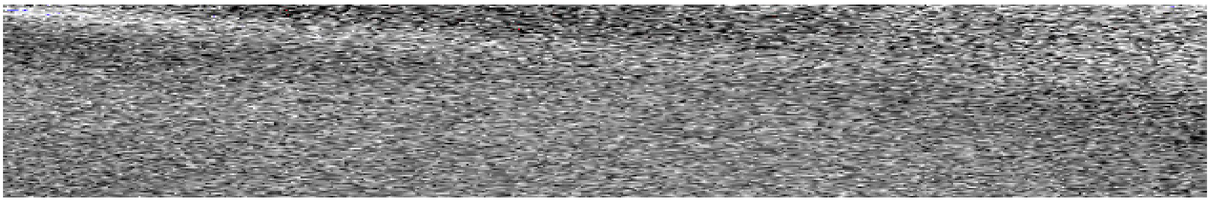

Figure S17: Residuals bitmap for the c(s) distribution fit to the AUC-SV data for the STIL-A722L-Q729L peptide ($\bar{v}$ = 0.751 cm^3 g^{-1}) at 10 µM shown in the Figure 3A. Data were recorded at 60 krpm returning fit parameter values of: sw = 1.483 S; sw(20,w) = 1.530 S; f/f0 =1.390; and mw 16134 = Da (4.2 × monomer mass) at 95% confidence level. Residuals are shown as a bitmap in which the greyscale shade indicates the difference between the fit and raw data, where black and white colors represent data that have extreme deviations from the fit.

Sedimentation equilibrium experiments

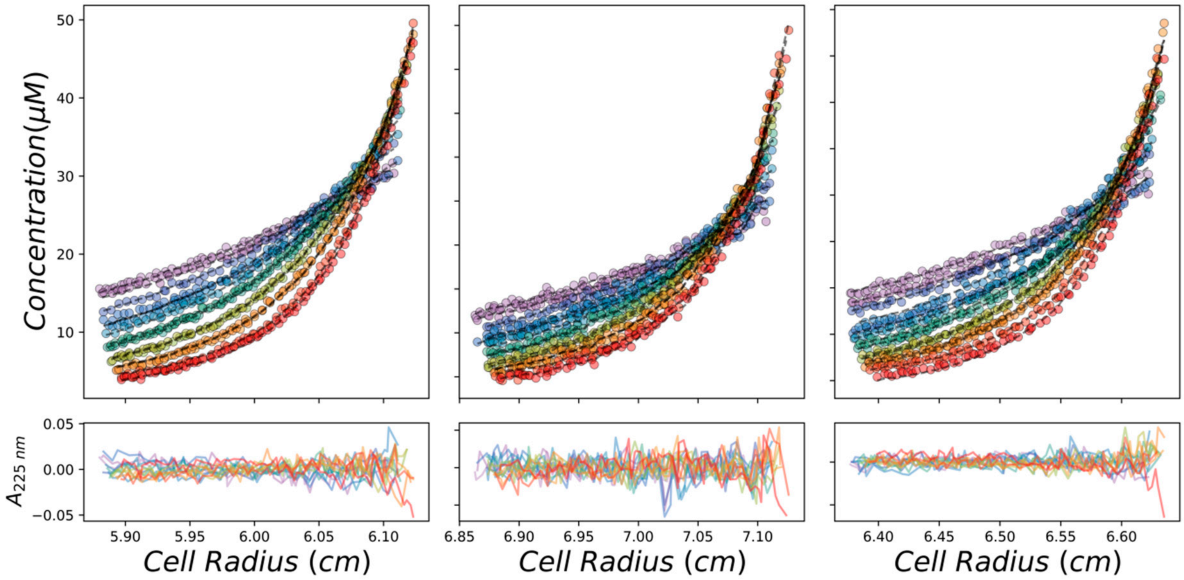

Figure S18: AUC-SE of WT STIL CCD at starting concentrations of (from left to right) 20, 5, and 10 μM. Plots show the concentration of the peptide (converted from the absorbance at 225 nm to using the relative molar extinction coefficient of the peptide) across the cell of the experiment. Raw data are shown as coloured circles (24 krpm, purple; 28 krpm blue; 32krpm, teal; 36krpm, yellow; 40krpm, orange; 48 krpm, red), and fits are shown as dashed black lines. Residuals between the raw data and the fit are shown in a separate plot below coloured by the same scheme as the plots above.

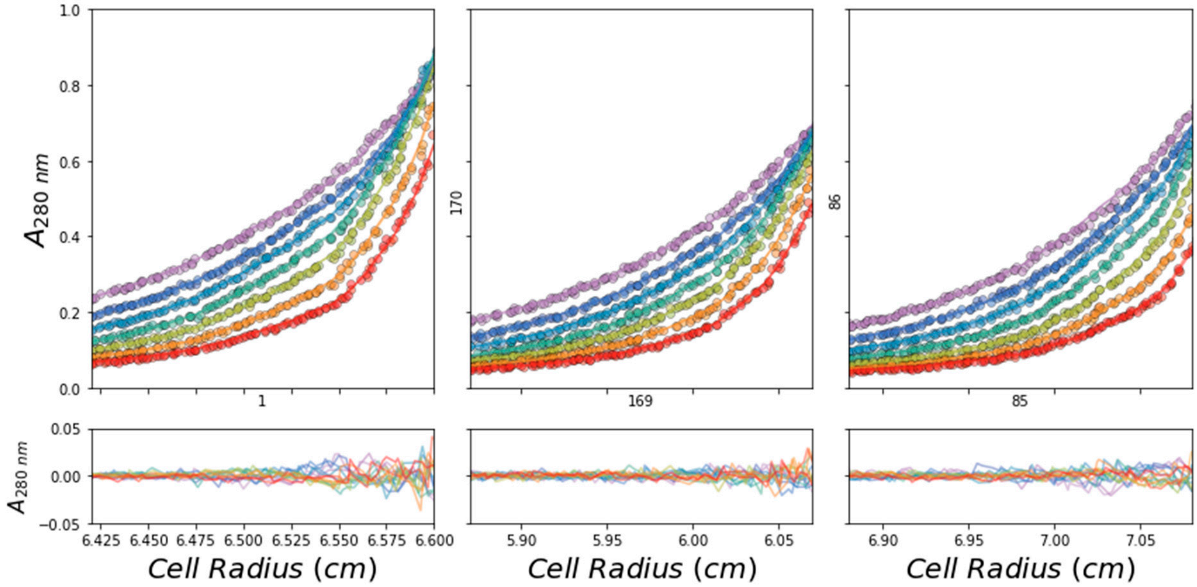

Figure S19: AUC-SE of STIL-WT at a starting concentration 500 μM in triplicate. Plots show the absorbance at 280 nm across the cell of the experiment. Raw data are shown as coloured circles (24 krpm, purple; 28 krpm blue; 32krpm, teal; 36krpm, yellow; 40krpm, orange; 48 krpm, red), and fits are shown as solid lines. Residuals between the raw data and the fit are shown in a separate plot below coloured by the same scheme as the plots above. The fit to a single species model returned a mass of 14315 ± 127 Da (3.8 × monomer mass, 95% confidence limits).

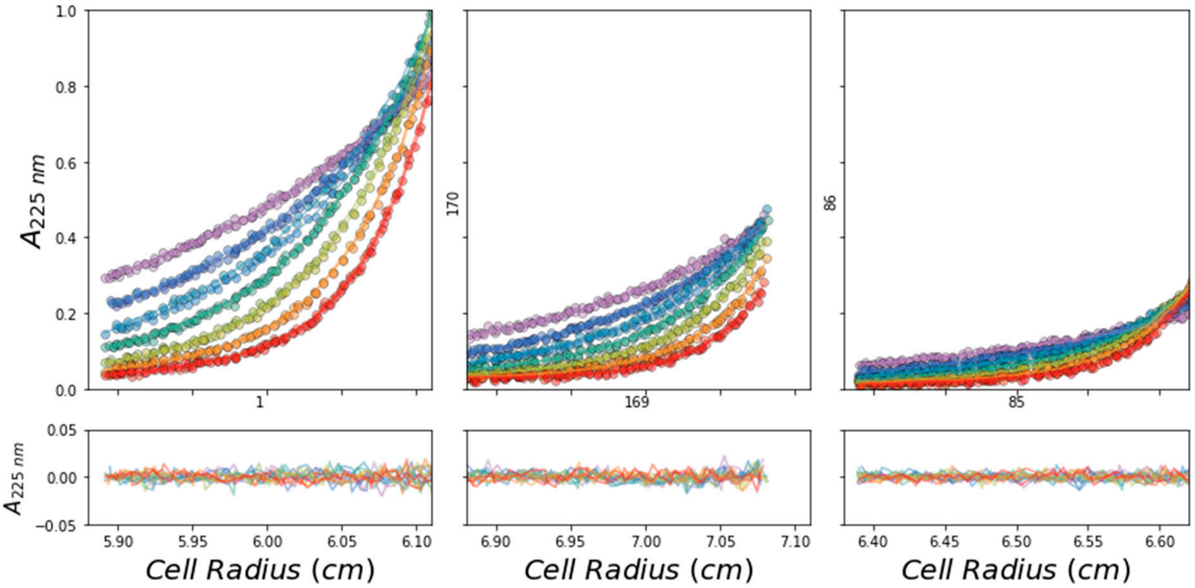

Figure S20: AUC-SE of STIL-A722L at starting concentrations of (from left to right) 20, 10, and 5 µM. Plots show the absorbance at 225 nm across the cell of the experiment. Raw data are shown as coloured circles (24 krpm, purple; 28 krpm blue; 32krpm, teal; 36krpm, yellow; 40krpm, orange; 48 krpm, red), and fits are shown as solid lines. Residuals between the raw data and the fit are shown in a separate plot below coloured by the same scheme as the plots above. The fit to a single species model returned a mass of 13541 ± 94 Da (3.5 × monomer mass, 95% confidence limits).

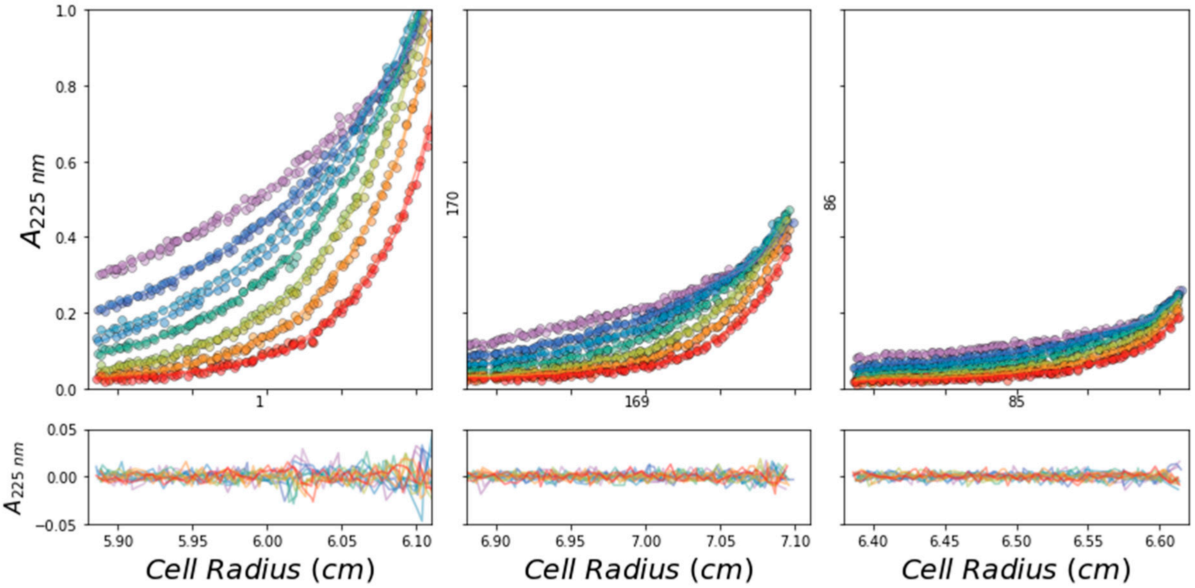

Figure S21: AUC-SE of STIL-Q722L at starting concentrations of (from left to right) 20, 10, and 5 µM. Plots show the absorbance at 225 nm across the cell of the experiment. Raw data are shown as coloured circles (24 krpm, purple; 28 krpm blue; 32krpm, teal; 36krpm, yellow; 40krpm, orange; 48 krpm, red), and fits are shown as solid lines. Residuals between the raw data and the fit are shown in a separate plot below coloured by the same scheme as the plots above. The fit to a single species model returned a mass of 13373 ± 200 Da (3.5 × monomer mass, 95% confidence limits).

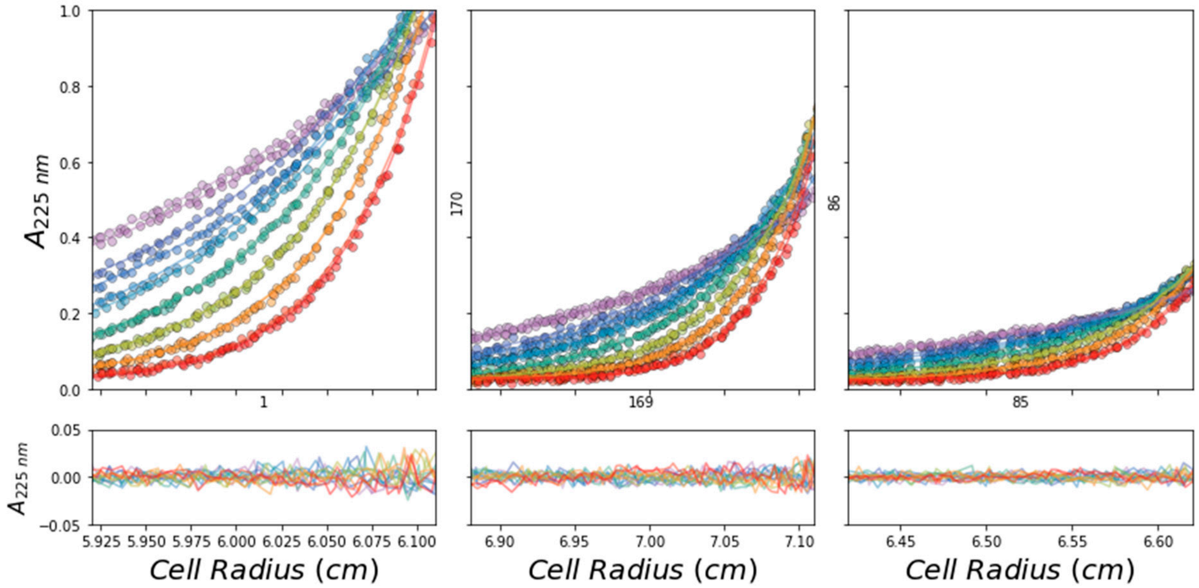

Figure S22: AUC-SE of STIL-A722L-Q722L at starting concentrations of (from left to right) 20, 10, and 5 µM. Plots show the absorbance at 225 nm across the cell of the experiment. Raw data are shown as coloured circles (24 krpm, purple; 28 krpm blue; 32krpm, teal; 36krpm, yellow; 40krpm, orange; 48 krpm, red), and fits are shown as solid lines. Residuals between the raw data and the fit are shown in a separate plot below coloured by the same scheme as the plots above. The fit to a single species model returned a mass of 12446 ± 129 Da (3.2 × monomer mass, 95% confidence limits).

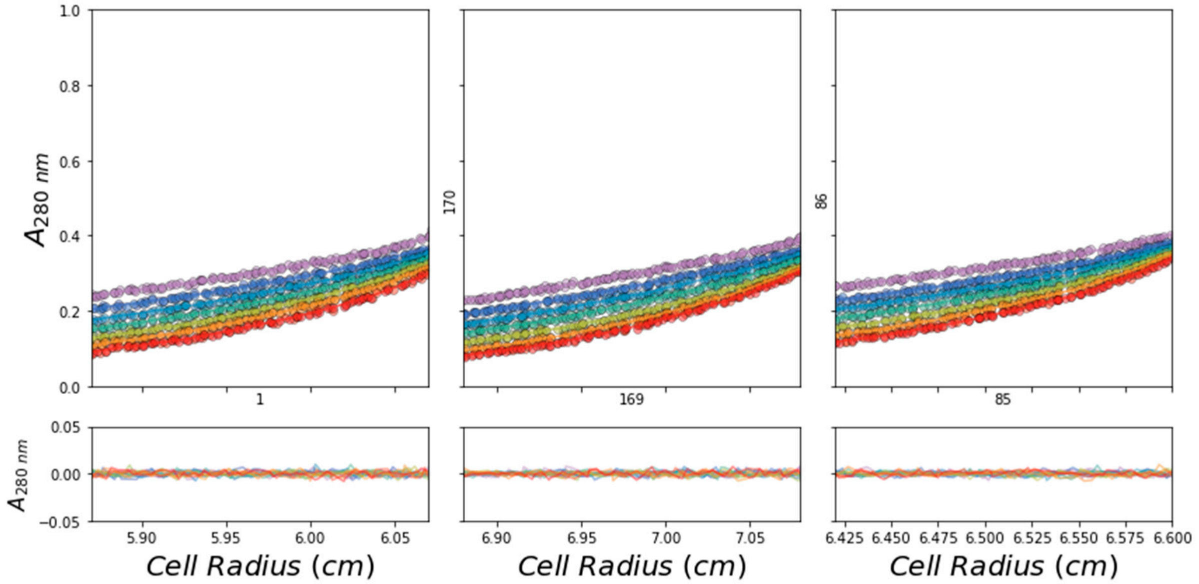

Figure S23: AUC-SE of STIL-L736E at a starting concentration 500 µM in triplicate. Plots show the absorbance at 280 nm across the cell of the experiment. Raw data are shown as coloured circles (24 krpm, purple; 28 krpm blue; 32krpm, teal; 36krpm, yellow; 40krpm, orange; 48 krpm, red), and fits are shown as solid lines. Residuals between the raw data and the fit are shown in a separate plot below coloured by the same scheme as the plots above. The fit to a single species model returned a mass of 3038 ± 292 Da (0.8 × monomer mass, 95% confidence limits).

Table S4: Summary of binding affinities for STIL CCD oligomerization as determined using different techniques in this study

	Measured dissociation constant (µM)	Technique
Dimerization	8 ± 2	Fluorescence, AUC-SV
Dimerization	8.5 ± 0.4	fluorescence polarization
Tetramerization	68 ± 2	AUC-SE

Section 4. Crystallography

Table S5: Statistics of the X-ray crystal structures of STIL-L736E and STIL-Q729L

	**STIL-CCD-L736E**	**STIL-CCD-Q729L**
PDB Accession Code	8OYK	8OYL
Wavelength (Å)	0.97	0.9999
Resolution range	63.83 – 1.90 (1.94-1.90)	31.46-1.92 (2.00-1.92)
Space group	P 1 21 1	I 2 2 2
Unit cell	43.54 31.27 64.47<br>90.00 98.08 90.00	24.97 34.94 72.32<br>90.00 90.00 90.00
Total reflections	25868 (1649)	16732 (1665)
Unique reflections	13477 (846)	2609 (280)
Multiplicity	1.9 (1.9)	6.4 (5.9)
Completeness (%)	97.7 (96.6)	99.9 (100.0)
Mean I/sigma(I)	15.4 (6.0)	7.1 (3.2)
Wilson B-factor	15.5	20.3
R-merge	0.038 (0.119)	0.14 (0.346)
R-meas	0.054 (0.168)	0.153 (0.380)
R-pim	0.038 (0.119)	0.08 (0.152)
CC1/2	0.996 (0.957)	0.989 (0.89)
Reflections used in refinement	12795	2338
Reflections used for R-free	680	270
R-work	0.180 (0.191)	0.149 (0.175)
R-free	0.225 (0.235)	0.190 (0.205)
Number of non-hydrogen atoms	1697	286
macromolecules	1591	268
ligands	1	6
solvent	105	12
Protein residues	186	32
RMS(bonds)	0.0093	0.012
RMS(angles)	1.39	1.39
Ramachandran favored (%)	100	100
Ramachandran allowed (%)	0.00	0.00
Ramachandran outliers (%)	0.00	0.00
Rotamer outliers (%)	1.18	0.00
Clashscore	2.8	4
Average B-factor	21	23
macromolecules	21.49	22.41
ligands	34.43	44.46
solvent	27.68	39.74
Number of TLS groups	6	1
Comercial Screen HT-96 ID	JCSG PlusTM, F3	Structure Screen 1 and 2, H2
Crystallisation Conditions	0.9 mM peptide, 50 mM Tris, and 10 % v/v MPD, at pH 8.0 and 4℃	1.5 mM peptide, 50 mM Cadmium chloride hemi(pentahydrate), 50 mM Sodium acetate, and 15 % v/v PEG 400, at pH 4.6 and 20℃
